# Mangroves as a major source of soil carbon storage in adjacent seagrass meadows

**DOI:** 10.1038/srep42406

**Published:** 2017-02-10

**Authors:** Guangcheng Chen, Muhammad Husni Azkab, Gail L. Chmura, Shunyang Chen, Pramudji Sastrosuwondo, Zhiyuan Ma, I. Wayan Eka Dharmawan, Xijie Yin, Bin Chen

**Affiliations:** 1 Third Institute of Oceanography, State Oceanic Administration, Xiamen, Fujian, China; 2 Research Centre for Oceanography, Indonesian Institute of Sciences, Indonesia; 3 Department of Geography, McGill University, Montreal, Quebec, Canada

## Abstract

Mangrove forests have the potential to export carbon to adjacent ecosystems but whether mangrove-derived organic carbon (OC) would enhance the soil OC storage in seagrass meadows adjacent to mangroves is unclear. In this study we examine the potential for the contribution of mangrove OC to seagrass soils on the coast of North Sulawesi, Indonesia. We found that seagrass meadows adjacent to mangroves had significantly higher soil OC concentrations, soil OC with lower δ ^13^C, and lower bulk density than those at the non-mangrove adjacent meadows. Soil OC storage to 30 cm depth ranged from 3.21 to 6.82 kg C m^−2^, and was also significantly higher at the mangrove adjacent meadows than those non-adjacent meadows. δ^13^C analyses revealed that mangrove OC contributed 34 to 83% to soil OC at the mangrove adjacent meadows. The δ^13^C value of seagrass plants was also different between the seagrasses adjacent to mangroves and those which were not, with lower values measured at the seagrasses adjacent to mangroves. Moreover, we found significant spatial variation in both soil OC concentration and storage, with values decreasing toward sea, and the contribution of mangrove-derived carbon also reduced with distance from the forest.

Although coastal vegetated wetlands have an area <2% of the ocean and one to two orders of magnitude smaller than terrestrial forests, these wetlands are ecologically important in global carbon cycling and sequestration^1^,^2^,^3^. Rates of organic carbon burial in marine coastal wetlands may contribute ~50% of that in global oceans^1^. Recent global syntheses estimated the carbon burial rate at 174 g C m^−2^ y^−1^ in mangrove forests^4^ and 160–186 g C m^−2^ yr^−1^ in seagrass meadow^5^.

The flooded, thus frequently anoxic conditions in wetland soils limits OC decomposition in soils, so storage is long-lived^6^,^7^. The coastal wetlands thus serve as globally significant carbon storage^8^,^9^. Seagrass meadows are one of the world’s most productive ecosystems and have been recognized as global hotspots for soil carbon storage (13.97 kg C m^−2^), twice as that of terrestrial soils^9^. However, regional assessments of carbon storage are still limited and needed to increase accuracy of the global budget. A survey from 17 Australian seagrass meadows revealed that soil carbon storage spatially varies with factors such as seagrass species and water depth^10^. Other studies have demonstrated the spatial variability of soil OC storage, with higher values nearshore than offshore within a seagrass meadow, which was attributed to variation in hydrodynamic properties, production and soil accumulation rates^11^.

Mangroves have been found to serve as important exporters of both organic and inorganic carbon^2^,^12^. Although a global extrapolation by Kennedy *et al*. suggested that ~50% of the surface soil carbon pool in seagrass meadows is authochthonous^13^, an extensive survey of the soil carbon sources in some southeast Asian seagrass meadows showed that their soil OC input are often dominated by mangrove organic matter^14^. However, Wooller *et al*. found the source of OC in a mangrove forest soil was primarily allochthonous and included seagrass material^15^. A recent study in the Arabian Gulf suggested in-welling of seagrass production balanced the out-welling of mangrove production and also affected the deposit grazers and filter feeders in that arid mangrove forest^16^. These findings suggest that the cross-ecosystem OC flux between mangrove and seagrass on could be geographically variable, and warrants further research.

The accumulation of mangrove-derived material in adjacent seagrass soils has been demonstrated through comparisons of OC concentrations^17^ and stable isotope evidence^14^ from seagrass meadows with varying distances to mangrove stands^18^,^19^,^20^. Although higher soil carbon concentration and storage at seagrass meadows can be attributed to the contributions from adjacent mangrove forest, evidence from seagrass meadows with varying distances to mangrove could not exclude the possibility that higher levels of carbon storage in seagrass meadows are due to their shore-proximal locations that can be associated with higher seagrass coverage and lower hydrodynamic energy which would stimulate the soil carbon burial^11^.

Southeast Asia is a major center of seagrass diversity^21^,^22^ and the quantitative studies on carbon storage in this area are important for the accuracy of global budget. In this study, we compared the soil carbon storages and their source between the mangrove adjacent seagrass meadows and non-mangrove meadows in North Sulawesi, Indonesia, to test whether mangrove-derived OC would enhance the soil OC storage at their adjacent seagrass meadows. Our study provided nearshore conditions that exclude mangroves, which serves as a control to confirm the supposition that it is indeed mangrove presence that results in increased C storage. The spatial variation of carbon storage/sources with distance offshore was also investigated at each seagrass meadow to test the extent of mangrove impact. We hypothesized that the seagrass meadows could be substantial carbon storage in this area, and the mangrove-derived OC dominates the carbon sources and could enhance the OC storage at the mangrove adjacent seagrass meadow. Moreover, spatial variability with distance offshore could be the feature of their carbon storage and the impact of mangrove decreases with its distance from seagrass meadow.

## Results

### Soil carbon content and carbon storage

**Figure 1:**
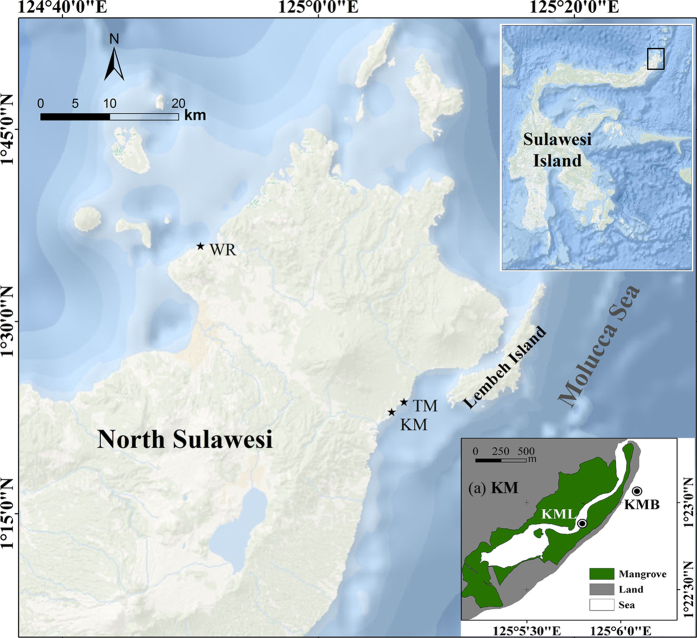
Locations of the four sampling sites in North Sulawesi, Indonesia. The map was created with ArcGIS version 10.0 (https://www.arcgis.com) using the World Ocean Base freely available at http://services.arcgisonline.com/ArcGIS/services. The map of Kema area (**a**) was created with ArcGIS version 10.0 using the digitizing Google Earth (Version 7.1, https://www.google.com/earth/) image (Image ©2016 DigitalGlobe). WR: Wori; TM: Tanjung Merah; KM: Kema; KML: Kema lagoon; KMB: Kema beach.

**Figure 2:**
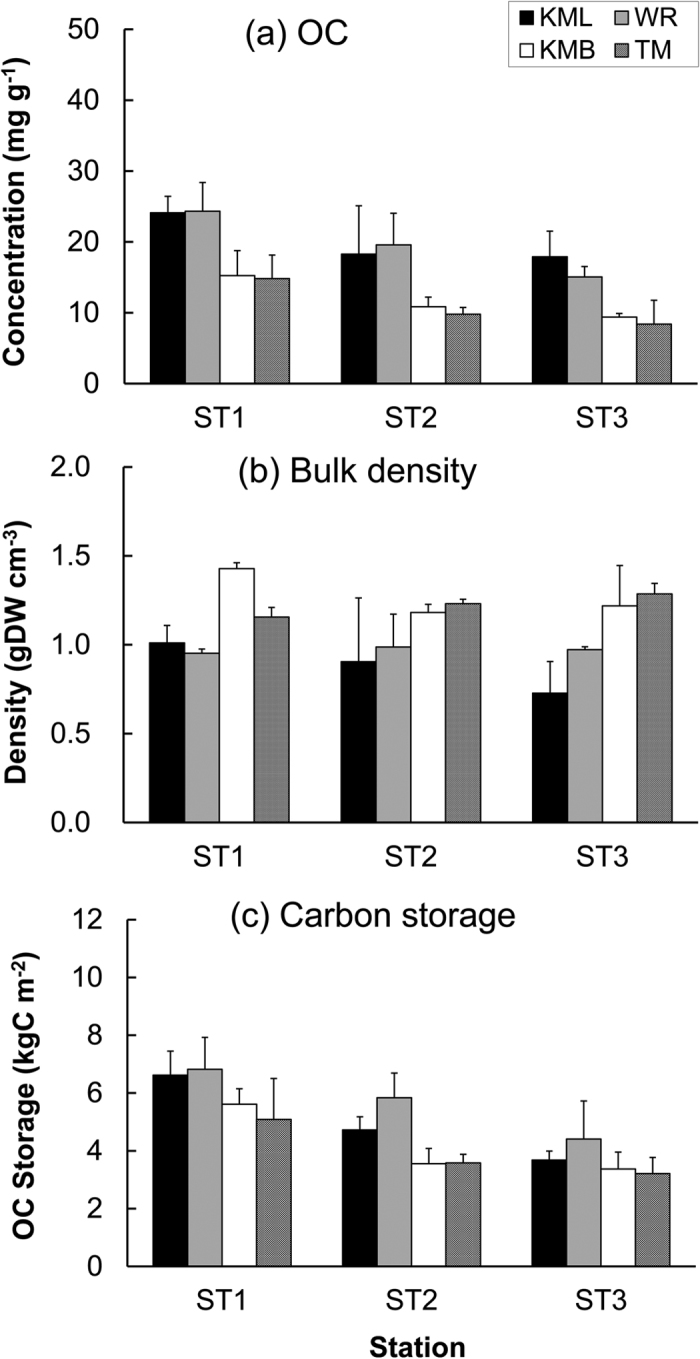
Soil organic carbon concentration (**a**), bulk density (**b**) and carbon storage (**c**) in the four seagrass meadows in North Sulawesi. KML: Kema lagoon; WR: Wori; KMB: Kema beach; TM: Tanjung Merah (mean and standard deviation of three replicates are shown). ST1 was the mangrove-fringed station (for WR and KML) or the landward station (for TM and KMB); ST3 was the off-shore station and ST2 was midpoint between ST1 and ST3.

The OC concentration in the surface soil (to a depth of 30 cm) in North Sulawesi (Fig. 1) differed among the four seagrass sites investigated and varied significantly with distance offshore (Table 1, Fig. 2a) according to the two-way ANOVA test. Soil OC concentrations at the two mangrove adjacent sites KML (17.1 ± 7.0 mg g^−1^ to 24.1 ± 2.3 mg g^−1^) and WR (15.1 ± 1.5 mg g^−1^ to 24.3 ± 4.0 mg g^−1^) were significantly higher than those at the non-mangrove adjacent sites (8.4 ± 3.4 mg g^−1^ to 15.2 ± 3.5 mg g^−1^), irrespectively of the sampling stations. OC concentrations at sites with similar habitats (i.e., WR vs. KML, TM vs. KMB) were not statistically different. At each set of the three seagrass sample stations located at varying distances from mangrove or land edge the OC concentrations of the two most offshore were not significantly different from each other, but were significantly lower than stations closest to the mangrove or land edge.

The bulk density was comparable among the sampling station (Table 1, Fig. 2b). Contrary to soil OC concentration, soil bulk density was higher at the two non-mangrove adjacent seagrass sites than the mangrove adjacent sites, and their respective values varied from 1.10 ± 0.14 to 1.43 ± 0.03 g cm^−3^ and from 0.73 ± 0.18 to 1.01 ± 0.10 g cm^−3^.

Soil OC storage was higher at the ST1 stations, with values ranging from 5.09 ± 1.41 to 6.82 ± 1.10 kg C m^−2^ in the surface soils to a depth of 30 cm (Table 1, Fig. 2c). Soil OC storage at the two offshore stations had similar values. The results indicated a similar variation trend to that of soil OC concentration. The mean OC storages over the three stations were 4.90 kg C m^−2^ and 5.68 kg C m^−2^ at the KML and WR sites, respectively, and were lower at the two non-mangrove adjacent sites (4.18 kg C m^−2^ at KMB and 3.96 kg C m^−2^ at TM).

### Plant organic carbon

**Figure 3:**
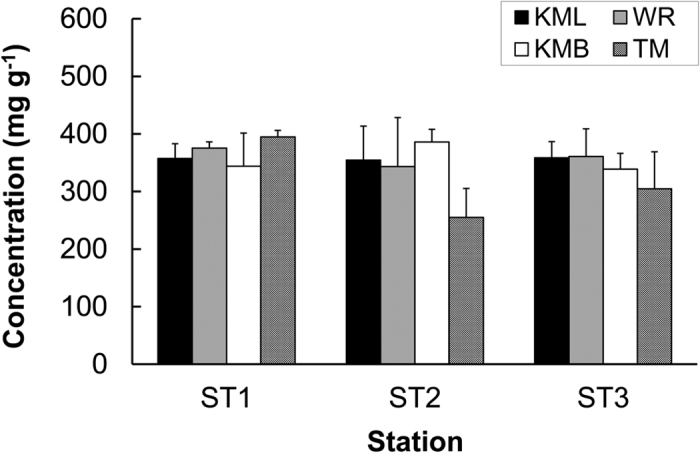
Organic carbon concentrations in seagrass plants in North Sulawesi (mean and standard deviation of three replicates are shown). Same abbreviations as Fig. 2.

Organic carbon concentration in the seagrass tissues was comparable among sites and among different stations, and ranged from 255 ± 50 mg g^−1^ to 386 ± 22 mg g^−1^ in this study (Table 1, Fig. 3). The OC concentrations were 443 ± 18 mg g^−1^ and 417 ± 10 mg g^−1^ in the leaves of *Bruguiera gymnorrhiza* and *Rhizophora apiculata*, respectively at WR mangrove forest, and the concentration was 408 ± 6 mg g^−1^ in *R. apiculata* leaves collected at KML, which were higher than those in seagrass tissues.

### Soil and plant δ^13^C

**Figure 4:**
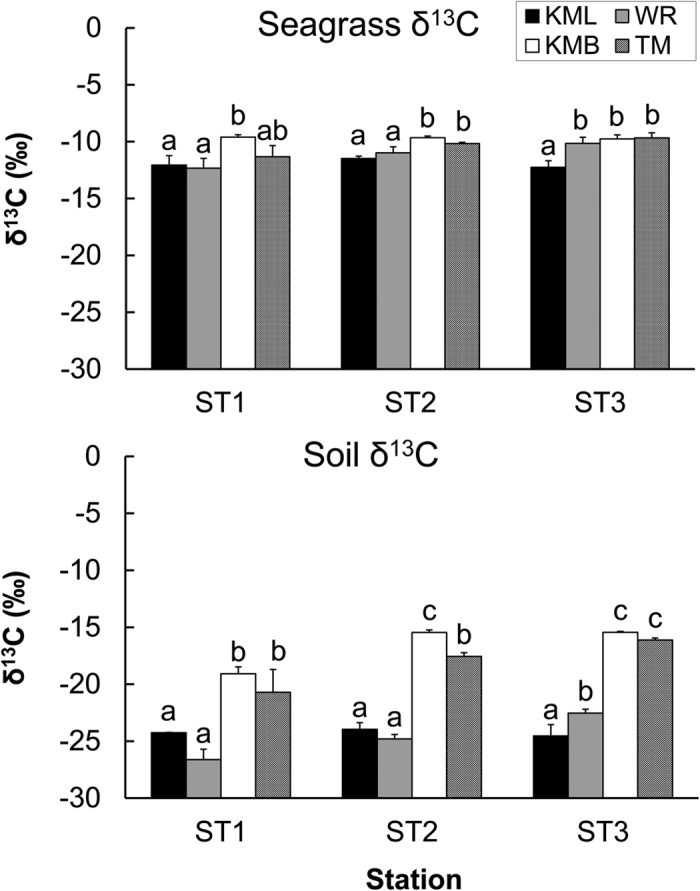
δ^13^C of seagrass (**a**) and soil organic carbon (**b**) in the four seagrass meadows in North Sulawesi (mean and standard deviation of three replicates are shown). Same abbreviations as Fig. 2. At each sampling station, different letters indicate significant difference among the four sites according to ANOVA test.

δ^13^C values of the mangrove leaves were −29.5 ± 0.2‰ and −29.6 ± 0.5‰ at the KML and WR mangrove forests, respectively. Seagrass tissues had δ^13^C values varying form −12.3 ± 0.9‰ to −9.6 ± 0.2‰ at the four sites (Fig. 4a), and the values also changed with the two factors (Table 1). There was no significant difference in δ^13^C with the distance offshore at the KML and KMB sites, but the OC was significantly depleted in ^13^C at the ST1 than the ST2 station at the other two sites. Seagrass OC at stations close to mangrove or shore generally was more ^13^C enriched at the non-mangrove adjacent sites than those at the mangrove adjacent sites.

The δ^13^C of soil carbon significantly varied with seagrass site and sampling station, but the spatial variation was site specific (Table 1 and Fig. 4b). The soil δ^13^C varied little among the three stations at KML, while it gradually increased from the ST1 to the ST3 station (i.e. seaward) at the WR site. Soil OC at the non-mangrove adjacent sites was more depleted in ^13^C at station ST1 than the other two stations. The soil δ^13^C at the mangrove adjacent sites (ranging from −26.6 ± 0.9‰ to −22.5 ± 0.3‰) was lower than those at the non-mangrove adjacent meadows (ranging from −20.7 ± 2.0‰ to −15.5 ± 0.1‰) and closer to the δ^13^C of mangrove litter than to the seagrass plant tissue.

### Source of the organic carbon in seagrass meadows

Organic carbon in the seagrass meadow soils consists of a mixture of different sources (Table 2). The contribution of mangrove-derived OC was greater than other sources at the mangrove adjacent seagrass meadows, and >70% of the soil OC at ST1 on the WR transect. Seagrass contributed <35% of soil OC and likely less than the epiphytes and suspended particulate organic matter (SPOM) at these two mangrove adjacent sites. At the WR site the contribution of mangrove OC was greatest at the ST1 station, and decreased gradually seaward.

At the two non-mangrove adjacent seagrass sites, the contribution of non-mangrove terrestrial OC to the soil was close to that of other sources at the ST1 station, and the proportion declined at the seaward stations. The contribution of seagrass was similar to that of epiphytes and SPOM and these three sources comprised the majority of the soil OC at the two seaward sites at the non-mangrove adjacent sites.

## Discussion

Previous studies have indicated that seagrass soils have significantly higher OC contents than adjacent unvegetated soils^13^,^23^. Studies which report organic matter in both seagrass soils and adjacent bare soils show that the seagrass soils contain 18–47 mg g^−1^ of organic matter; whereas the concentration is lower in the adjacent bare soils varying from 0.7 to 22 mg g^−1^ 23. The global average OC concentration in seagrass soil was estimated as 25 mg g^−1^ 9. The range of OC concentrations in the North Sulawesi seagrass meadow soils (8.4 to 24.3 mg g^−1^) falls within the global range (0 to 480 mg g^−1^)^9^.

The OC storage in the surface soil (to a depth of 30 cm) ranged from 3.21 to 6.82 kg C m^−2^ at the four seagrass meadows in North Sulawesi. As the sampling was limited to the surface soil, it is difficult to directly compare the soil OC storage in this study with those down to 1 m^11^,^24^. However, our results are higher than those measured at seagrass meadows to a similar depth in Australia (0.26 to 4.83 kg C m^−2^ in the top 25 cm)^10^ and are comparable to the storage at the top 50 cm seagrass soil in Abu Dhabi, United Arab Emirates^25^. The soil OC concentrations in our study are higher than those reported elsewhere in Southeast Asia (2 to 17 mg g^−1^)^14^ further confirming that seagrass meadows in North Sulawesi are rich in soil OC and are sites of substantial carbon storage. Alongi *et al*. reported that the OC storage in the surface 1 m of Indonesian seagrass meadows ranged from 3.13 to 29.33 kg C m^−2^, and suggested that Indonesian seagrass and mangroves account for 17% of the global blue carbon reservoir^24^.

Organic carbon in seagrass soil is potentially derived from seagrass, phytoplankton, epiphytes, and mangrove/terrestrial organic matter^13^,^14^. Previous studies based on δ^13^C analyses of soil OC have recognized seagrass tissue as the major contributor to soil carbon pool under the seagrass canopy^26^, and globally it is estimated that seagrass derived organic matter contributes ~50% of the carbon in the surface soil of the seagrass meadows^13^. However, Kennedy *et al*. suggest that where seagrass meadows are located in enclosed bays or close to mangroves, allochthonous organic matter may increase in importance^13^ and the δ^13^C of some Southeast Asian seagrass meadow soils is much lower than the δ^13^C of seagrass tissue reflecting the contribution of allochthonous sources such as mangrove OC, epiphyte OC and SPOM^14^. Similar to these Southeast Asian meadows, in our study seagrass tissue contributed a minimal proportion of the soil OC in the meadows adjacent to mangrove forests. Even in the two non-mangrove adjacent meadows, seagrass was rarely the dominant OC source in the underlying seagrass soils. These results suggest that the role that seagrass plays is geographically variable. Some studies also demonstrated that the contribution of seagrass to food webs or net ecosystem metabolism is generally less^17^,^27^,^28^ and microalgae including epiphytes also contribute a large proportion of the total OC production in flora communities^19^,^29^ and the trophic importance^30^.

Organic matter produced by mangroves may accumulate in local soils or be transported to adjacent coastal waters as detritus^31^. Adame and Lovelock^32^ found that litter export from mangrove forests averages ~200 g C m^−2^ y^−1^, roughly accounting for 50% of the average C production in litterfall. It seems that the exportation of mangrove primary production could be more relevant than seagrass to the OC burial as only 30 to 50% of the seagrass net primary production (120 g C m^−2^ yr^−1^) is buried in seagrass meadows, i.e. the buried seagrass OC is less than 60 g C m^−2^ yr^−1^ in local soil^33^. Some studies in the seagrass meadows located adjacent to mangrove forest revealed that mangrove organic matter often dominates the soil input of OC^14^,^20^. Isotope mixing calculations in this study showed that mangrove-derived OC was the major source of soil OC in seagrass meadows adjacent to mangrove in the North Sulawesi and the contribution could also be substantial at the offshore stations (>100 m from the edge of the mangrove forest), indicating the importance of mangrove-derived OC in the carbon deposition and storage in their adjacent seagrass meadows. The buried mangrove OC in seagrass meadows then could provide substrate for soil bacterial communities^20^ and consumers at higher trophic levels^34^. On this basis, the mangrove/terrestrial carbon should also be taken into account when identifying the primary carbon source of the consumers in the seagrass meadows, particularly in those adjacent to mangrove stands.

As waves propagate over seagrass meadows, wave energy and current velocity are reduced, and seagrass therefore enhances the deposition of organic matter through trapping seston particles by the seagrass leaves and reducing particle-carrying capacity of the water^35^,^36^,^37^,^38^,^39^,^40^. The reduced wave energy and current velocity at the seagrass station proximal to the land edge thus favor the particulate OC deposition and the development of seagrass^36^. The denser seagrass canopy observed at the ST1 station in this study likely is due to the increased trapping of organic material as a source of nutrients^39^ and could further enhance the trapping of organic matter within the meadow. These features may result in the significant spatial variations in soil OC concentration and storage found in this study, which were higher at the seagrass meadows adjacent to mangrove or land edge. Serrano *et al*. found higher soil OC concentration/storage and proportion of fine particle material at the meadows close to the upper distribution limit, which was related to the input of seagrass material^11^. The results further suggest that such spatial variation is a common feature of seagrass meadows and together with the biogeochemical factors affecting soil OC storage^11^ should be taken into account for more accurate evaluations of carbon storage in seagrass meadows.

**Figure 5:**
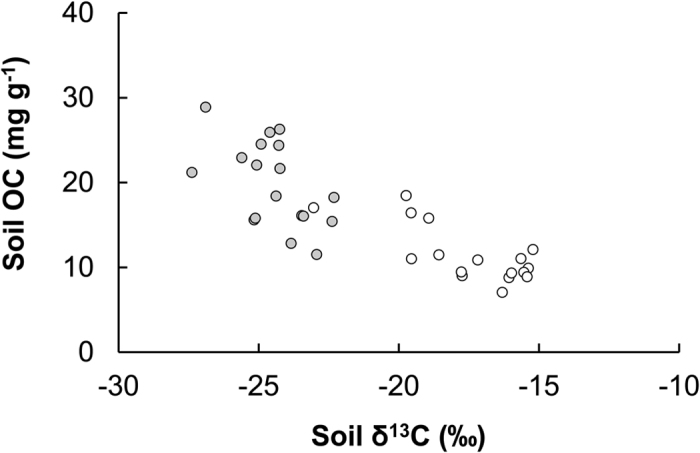
The relationships between the concentration and δ^13^C of soil OC in seagrass meadows (n = 36). White symbols: data from non-mangrove adjacent seagrass meadows; grey symbols: data from mangrove adjacent seagrass meadows.

Mangrove leaves had higher OC concentrations than the seagrass tissues in this study and the mangrove soils in North Sulawesi are more enriched in OC, ranging from 31 to 130 mg g^−1^ 41, compared to the seagrass soils in this study. The exported OC in the form of mangrove detritus could be efficiently trapped in the dense seagrass meadows adjacent to mangroves and result in a higher soil OC concentration at the interface between mangrove and seagrass. The relationship between soil OC concentrations and δ^13^C values (Fig. 5) indicates that soil OC concentration changes as a function of OC accumulation in the soil and the increase in soil OC concentration is due to the input of ^13^C-depleted organic matter from the mangrove. This supply of mangrove carbon declines with distance from the forest^18^,^19^,^42^. The estimated fractional contribution in WR also clearly showed the spatial pattern of with the mangrove C contribution. However, this accumulation of isotopically light mangrove detritus is generally accompanied by lower soil bulk density at the mangrove adjacent meadows so that the enhancement of soil OC storage by the mangrove-derived OC in the seagrass meadow is not as apparent as that of soil OC concentration. The present study measured the OC storage in the top 30 cm of seagrass soils and the OC storage at each seagrass site was higher at the mangrove adjacent meadows (Table 1, Fig. 1c). The KML site was located on a the tidal channel entering the lagoon the hydraulic energy was reduced compared to the open sea area and likely enhanced carbon burial within the lagoon. Therefore, the difference in soil OC storage between KML and open sea sites (KMB and TM) singly could not reflect the enhancement effect of mangrove on soil OC storage in the adjacent seagrass meadow. However, the isotope mixing calculations showed that mangrove OC contributed 45–71% of the soil OC at the KML seagrass meadow (Table 2) demonstrating that mangrove-derived OC contributed to the soil carbon storage at KML and the results indicated that mangrove derived OC enhances the soil OC storage at the adjacent seagrass meadows under both lagoon and open sea environments in this study. We further conclude that mangrove OC makes a substantial contribution to the soil OC concentration and storage in the upper 1 m as the soil OC profiles in Sulawesi seagrass meadows showed the consistently higher soil OC concentrations throughout the soil profile of a seagrass meadow affected by the mangrove out-welling than at meadows with less impact^24^.

The spatial variation of soil OC storage in this study was consistent with that found in previous studies reporting the carbon burial rates^5^,^11^,^14^,^43^. The seagrass meadows close to the sand banks accumulate soil OC storage at higher rates than those at the offshore meadows^11^, consistent with our finding that the soil OC storage tend to be higher at the meadows adjacent to mangrove or land edge. Seagrass meadows located around the sand banks also have strong carbon burial capacity^5^. A *Posidonia* meadow in northeast Spain had an annual deposition rate of carbon at 198 g C m^−2^ yr^−1^ 43, comparable to the rate reported for meadows adjacent to extensive mangroves in south Asia using the same sediment trap method^14^. These suggest that the carbon burial can be substantial at the meadows at ST1 (proximal to shore) stations in this study and it is reasonable that the soil OC storage at the ST1 station was comparable between the mangrove adjacent sites and the non-adjacent sites. Further researches on carbon storage and burial rates over different time scales and reporting the thickness of sediment deposits are needed to address the gaps in our understanding of the role of seagrass meadows as carbon sinks^5^. Rozaimi *et al*. also recommended to access and compare the OC storage among seagrass meadows based on the normalized data by period of accumulation^44^.

The δ^13^C values of seagrass measured in this study were within the range reported in other studies (−20.8 to −5.9‰)^45^. The δ^13^C of mangroves are more depleted than (<−24‰)^18^,^46^,^47^ than that of seagrasses. The OC of seagrass tissues in our study was more isotopically depleted at meadows adjacent to mangroves, indicating that the occurrence of mangroves also affects the isotopic composition of seagrass. One probable explanation is that seagrass could utilize the inorganic carbon derived from mangrove that is more depleted in ^13^C. This is reasonable as mangrove leaf material shows little change in the δ^13^C signal during decomposition^48^ and the inorganic carbon exported due to respiratory CO_2_ during decomposition of mangrove OC is likely to have a depleted δ^13^C. Previous studies have also suggested that ^13^C of seagrasses adjacent to mangrove forests become more enriched with increasing distance from the mangrove and that δ^13^C-depleted inorganic carbon derived from the decomposition of mangrove is exported in the tidal water and contributes to the low δ^13^C values of the seagrasses after assimilation^18^,^47^. On basis of the results in previous and the present studies, we suggested that seagrass meadows play important role in limiting the exportation of both mangrove-derived organic and inorganic carbon toward the coastal ocean, which has also been proposed by Bouillon *et al*.^42^. However, the information on the utilization of mangrove-derived inorganic carbon and its implication to the dynamics of mangrove carbon, as well as the involvement of seagrass species in this process are still limited, and deserve further investigation.

## Methods

### Study area

The province of North Sulawesi has a typical equatorial climate and the mean temperatures at sea level are uniform, varying only from 20 to 28 °C throughout the region and throughout the year. Tides in this area are mixed and mainly semi-diurnal, and fluctuate slightly with an annual tidal range of 2.4 m. The study area is subjected to strong influences of two monsoons, the wet northwest monsoon from November to March and the dry southeast monsoon from May to September^49^.

Four nearshore sub-tidal seagrass meadows were selected (Fig. 1), including two located at Kema (KM, 1°22′56.47″N, 125° 5′43.65″E), one at Tanjung Merah (TM, 1°23′43.78″N, 125°6′41.64″E) and one at Wori (WR, 1°35′54.46″N, 124°50′47.59″E). The TM seagrass lies along the sandy beach and is dominated by *Halodule pinifolia, Cymodocea rotundata* and *Thalassia hemprichii*. The WR site is situated between the mangrove forest and fringing coral reef, with the same seagrass species as at TM. At KM, sampling was carried out at a sandy-beach adjacent to a seagrass meadow on the open sea (KMB) and a meadow located at the tidal channel of the lagoon area (KML). The latter is adjacent to mangroves. The dominant seagrasses at KM are *Enhalus acoroides* and *T. hemprichii*. These four meadows are therefore representative for the mangrove adjacent (WR and KML) and non-mangrove adjacent (TM and KMB) seagrass meadows.

### Plant and soil samplings

At each of the four sites, three sampling stations, ST1, ST2 and ST3 with different distances offshore were established. The lengths of the transects from the land or mangrove edge were ~30 m, 130 m, 110 m and 110 m at the KML, WR, KMB and TM, respectively. ST1 was designated the mangrove-edge station at WR and KML, and the landward station at TM and KMB. ST3 signified the seaward zone at WR, TM and KMB sites. At KML ST3 was ~30 m from the mangrove edge and the longest distance from the mangrove in the sampling area. The sample stations “ST2” was located at the midpoint between the ST1 and ST3 stations.

Samples of vegetation (leaves, roots and rhizomes) were collected in three replicate quadrats (625 cm^2^) and taken back to laboratory. The plant tissues were cleaned of epiphytes and sand, and dried at 60 °C for 24 h. Many studies^13^,^14^,^20^,^46^ have used leaf material to represent seagrass-derived carbon. Instead, we combined the whole plant for δ^13^C analysis to reduce bias in the OC analysis, as a large fraction of plant biomass occurs as rhizomes and roots buried in the soil and contribute to the soil OC^50^. Moreover, Kennedy *et al*. also concluded that the belowground tissues had slightly enriched δ^13^C values than those of seagrass leaves (<1.5‰)^13^. Therefore, we considered that extrapolated OC sources from δ^13^C of the complete plant could be comparable to other studies using leaf material. Senescent mangrove leaves (with yellowish color) of the dominant species (*R. apiculata* and *B. gymnorrhiza* at WR, and *R. apiculata* at KML) were also collected at the sea-edge locations at KM and WR mangroves by gently shaking the branches.

Soil was collected using PVC tubes (inner diameter 70 mm), with metal cutters at their bottom edges. Tubes were manually inserted into the soil by gently turning the tube until a depth of 20–30 cm was reached at each sampling plot. In the laboratory, the soils were extruded by inserting a plunger at the bottom of the cores and carefully drawing the PVC liner down over the plunger. The soil cores were sub-sectioned at 10 cm intervals. Each subsection was weighed and then sliced into two halves, with one half oven-dried at 60 °C to determine the water content of fresh samples. Another half was then air-dried after removing the visible animals, plant residues and stones (>2 mm). Before grinding, the air-dried samples were subdivided into five subsamples using the fractional shoveling method^51^ and one of these subsamples was selected at random for later analysis.

### Sample analysis

The OC and δ^13^C in soil and plant samples were measured using a Thermo Flash EA 1112 HT- Delta V Advantages system. For isotopic analysis of soils, air-dried subsamples were placed into silver cups; acidified with diluted HCl (5%) and then oven-dried at 40 °C to remove the carbonates. OC and δ^13^C analysis of seagrass and mangrove tissues followed the same procedure, but mangrove leaves were not treated with HCl as no carbonate is expected to be present^20^. The stable carbon isotopic composition is reported in the δ notation as the ratio of the heavy to the light stable isotope in the sample (R_sample_) relative to that of a standard (R_standard_), i.e., δ_sample_ = 1000 [(R_sample_/R_standard_)−1], with standard = Vienna Pee Dee Bellemnite (VPDB) and R = ^13^C/^12^C. The reproducibility of OC and stable isotopic analysis were <0.6% and <0.2‰, respectively.

### Statistical analysis and estimation of organic matter sources

As the profiles of soil OC, carbon storage and δ^13^C (Supplementary Figs S1–3) were not the focus of this study, only the averages of entire soil core were used here. For those tubes (8 tubes) that the sampling depth was less than 30 cm, the carbon storage in the top 20 cm was estimated and then normalized to the top 30 cm of soil for comparability. The normality of variables was checked using Kolmogorov–Smirnov test. The results showed that the distribution patterns of all soil parameters were distributed normally (p > 0.05), so no transformation of data was performed. Two-way analysis of variance (ANOVA) was used to test for differences in soil and seagrass parameters among the seagrass sites and sampling stations. If the difference was significant (p < 0.05), a post-hoc Tukey test was used to determine the difference. All statistical analyses were performed using SPSS 17.0 for Windows (SPSS Inc. USA).

To determine the relative contribution of different sources to seagrass soil OC, the IsoSource software^52^ was used to examine all possible combinations of solutions for four potential sources, seagrass, mangrove/terrestrial OC, phytoplankton and epiphytes. At the two non-mangrove adjacent seagrass meadows, non-mangrove terrestrial vegetation occurred at the landward beach and its relative contribution to the OC in seagrass soil was also considered. The IsoSource software provides the ranges of source proportional contributions to a mixture when the number of sources is too large to permit a unique solution (>number of isotope systems +1)^52^. All combinations of these source terms were used to solve for their likely contribution to the sediments at 1% increment and 0.1‰ resolution. According to Du *et al*., in this area the δ^13^C values of epiphytes and phytoplankton collected as SPOM were −14.5 ± 1.8‰ and −19.5 ± 0.8‰, respectively^53^. As the δ^13^C values of mangrove leaves were similar between the two sites, we used their mean value for examination of sources at the two mangrove adjacent sites. For the non-mangrove adjacent meadows, we used the δ^13^C value of mangrove leaves to represent the terrestrial organic matter as these two categories have similar isotopic composition^13^,^54^.

## Additional Information

**How to cite this article:** Chen, G. *et al*. Mangroves as a major source of soil carbon storage in adjacent seagrass meadows. *Sci. Rep.* **7**, 42406; doi: 10.1038/srep42406 (2017).

**Publisher's note:** Springer Nature remains neutral with regard to jurisdictional claims in published maps and institutional affiliations.

## Supplementary Material

Supplementary Information

## Figures and Tables

**Table 1:** F values of two-way ANOVAs test showing the variations of soil and plant parameter with seagrass sites and sampling stations.

Parameter	Source of variation		
	Site	Station	Interaction
Soil OC	18.47^***^	13.16^***^	0.26
Soil bulk density	14.94^***^	1.04	1.58
Soil OC storage	8.00^**^	26.22^***^	0.85
Seagrass OC	1.63	1.74	2.18
Soil δ^13^C	261.03^***^	49.79^***^	7.60^***^
Seagrass δ^13^C	27.75^***^	8.74^**^	3.88^**^

*,** and *** indicate significant r value at P < 005, 001 and 0001, respectively.

**Table 2:** Ranges of proportional contributions of the four potential sources to the soil organic carbon in North Sulawesi seagrass meadows.

		Sources (%)			
Station		Mangrove/terrestrial	Seagrass	Epiphytes	SPOM
KML	ST1	49–69	1–28	0–34	0–48
	ST2	45–69	0–30	0–37	0–54
	ST3	51–71	1–28	0–31	0–48
WR	ST1	71–83	0–17	0–17	0–29
	ST2	55–74	1–25	0–29	0–42
	ST3	34–63	2–34	0–44	0–62
KMB	ST1	16–47	8–52	0–59	0–63
	ST2	0–29	0–70	0–93	0–59
	ST3	0–28	0–70	0–93	0–58
TM	ST1	13–51	0–46	0–58	0–86
	ST2	0–37	0–60	0–79	0–78
	ST3	0–32	0–67	0–84	0–54

SPOM: suspended particulate organic matter.
