# Supplementary material for: Mangroves as a major source of soil carbon storage in adjacent seagrass meadows
Source: Sci Rep. 2017 Feb 10;7:42406. doi: 10.1038/srep42406 (PMC5301194; doi:10.1038/srep42406)
Supplement: Supplementary Information [file srep42406-s1.pdf]

# Mangroves as a major source of soil carbon storage in adjacent seagrass meadows

Guangcheng Chen[a], Muhammad Husni Azkab[b], Gail L. Chmura[c], Shunyang Chen[a], Pramudji Sastrosuwondo[b], Zhiyuan Ma[a], I Wayan Eka Dharmawan[b], Xijie Yin[a], Bin Chen[a]

[a]*Third Institute of Oceanography, State Oceanic Administration, Xiamen, Fujian, China*

[b]*Research Centre for Oceanography, Indonesian Institute of Sciences, Indonesia*

[c]*Department of Geography, McGill University, Montreal, Quebec, Canada*

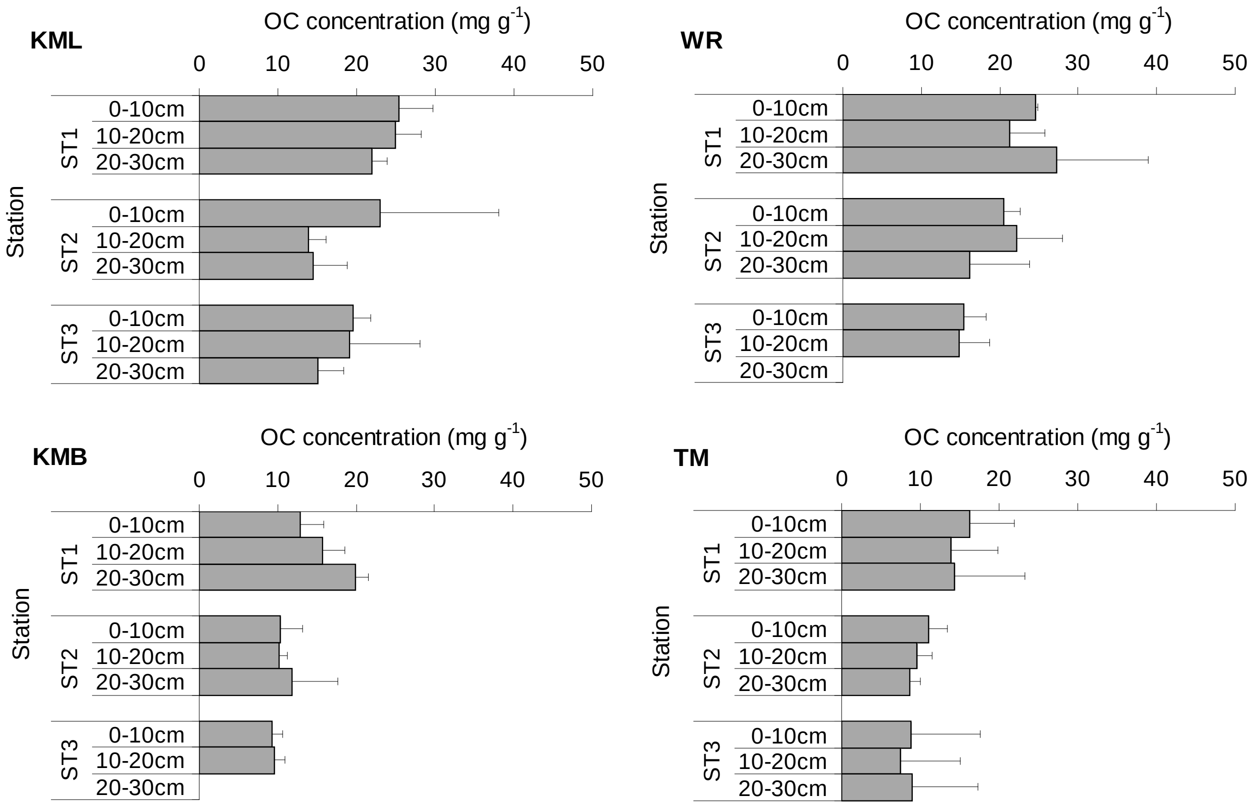


Supplementary Figure S1. Distribution of the soil organic carbon (OC) concentration in different soil depth sections at the four seagrass meadows. KML: Kema lagoon; WR: Wori; KMB: Kema beach; TM: Tanjung Merah (mean and standard deviation of three replicates are shown). ST1 was the mangrove-fringed station (for WR and KML) or the landward station (for TM and KMB); ST3 was the off-shore station and ST2 was midpoint between ST1 and ST3. Data of 20-30cm sections at the ST3 stations of WR and KMB sites are not available as the sampling depth were limited to 20cm.

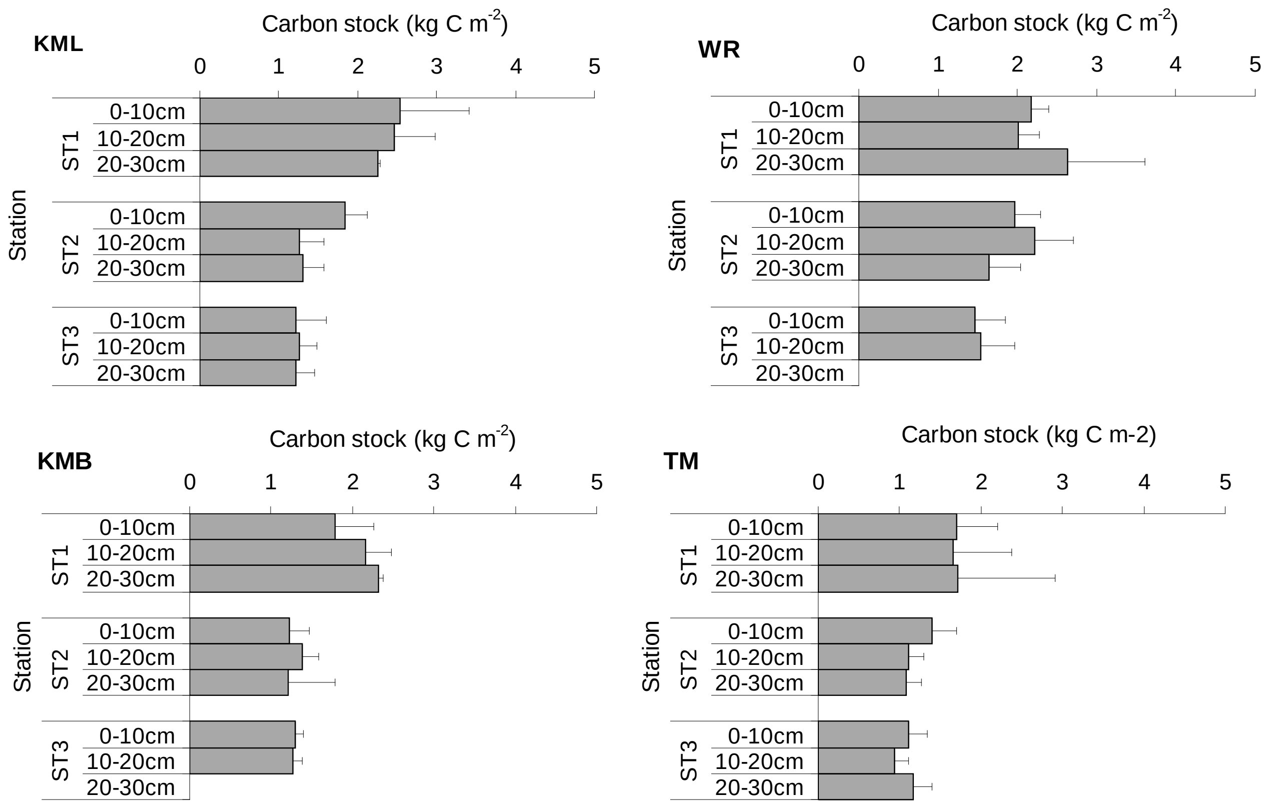


Supplementary Figure S2. Distribution of the soil organic carbon stock in different soil depth sections at the four seagrass meadows in North Sulawesi, Indonesia (mean and standard deviation of three replicates are shown). Same abbreviations as Supplementary Figure 1. Data of 20-30cm sections at the ST3 stations of WR and KMB sites are not available as the sampling depth were limited to 20cm.

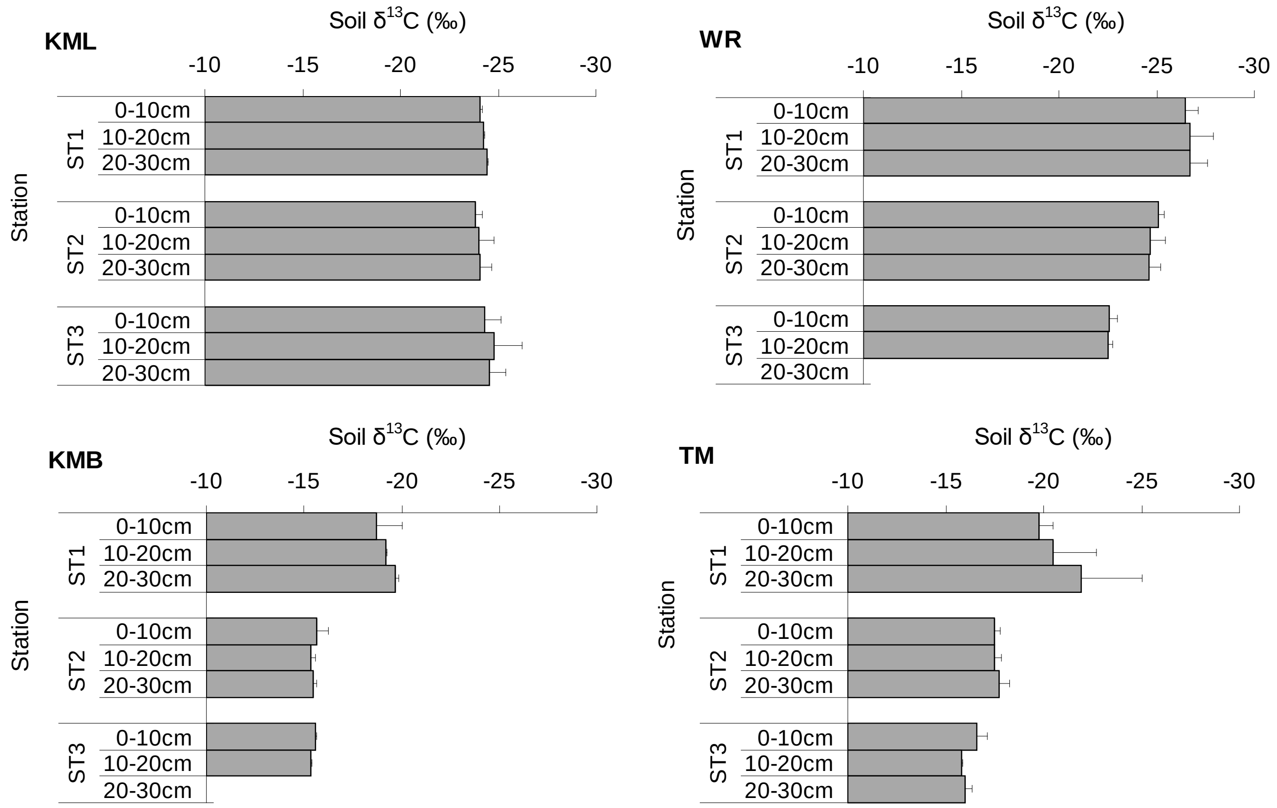


Supplementary Figure S3. Distribution of $\delta^{13}C$ of soil organic carbon in different soil depth sections at the four seagrass meadows in North Sulawesi, Indonesia (mean and standard deviation of three replicates are shown). Same abbreviations as Supplementary Figure 1. Data of 20-30cm sections at the ST3 stations of WR and KMB sites are not available as the sampling depth were limited to 20cm.
